# Using Positive Deviance to reduce medication errors in a tertiary care hospital

**DOI:** 10.1186/s40360-016-0082-9

**Published:** 2016-08-07

**Authors:** Fabio Teixeira Ferracini, Alexandre R. Marra, Claudio Schvartsman, Oscar F. Pavão dos Santos, Elivane da Silva Victor, Neila Maria Marques Negrini, Wladimir Mendes Borges Filho, Michael B. Edmond

**Affiliations:** 1 Pharmacy, Hospital Israelita Albert Einstein, São Paulo, Brazil; 2 Division of Medical Practice, Hospital Israelita Albert Einstein, Avenida Albert Einstein, 627 – bloco A1, 1° andar, sala 108, Morumbi, São Paulo, Brazil 05651-901; 3 Department of Internal Medicine, University of Iowa Carver College of Medicine, Iowa City, IA USA; 4 Statistics Department, Instituto Israelita de Ensino e Pesquisa (IIEP), Hospital Israelita Albert Einstein, São Paulo, Brazil; 5 Board of Healthcare Practice, Quality, Safety, and Environment, Hospital Israelita Albert Einstein, São Paulo, Brazil

**Keywords:** Patient safety, Medication errors, Prevention

## Abstract

**Background:**

The number of medication errors occurring in healthcare is large and many are preventable. To analyze medication errors and evaluate whether Positive Deviance is effective in reducing them.

**Methods:**

The study was divided into three phases: (2011- Phase I, control period; 2012 - Phase II, manager intervention, and 2013 - Phase III, frontline healthcare worker intervention). In Phases II and III, the Positive Deviance method (PD) was used to mitigate medication errors classified as “C” and higher according to the National Coordinating Council for Medication Error Reporting and Prevention (NCC MERP). The errors reported were compared across the three study phases, as well as by the location of the hospital unit, shift, cause, consequence, and the professional associated with the error.

**Results:**

A total of 4013 reported medication errors were analyzed. The largest number of errors occurred at the time the medications were administered, accounting for 35.5 % of errors in Phase I; 43.1 % in Phase II, and 55.6 % in Phase III. Nursing staff was most commonly associated with errors; 46.4 % of errors in Phase I, 48.5 % in Phase II, and 58.7 % in Phase III. With each intervention, a decrease was observed in the reported error rate of 0.12 (CI 95 %, 0.18 to 0.07).

**Conclusion:**

Positive Deviance proved to be effective, primarily when healthcare professionals who were involved in errors participated, as was observed in Phase III of this study.

## Background

The introduction of new diagnostic and therapeutic technologies has been associated with a parallel rise in adverse events. These errors are common, relevant, costly, and yet are avoidable [1]. In the US, studies indicate that medication errors account for at least one death per day and affect approximately 1.3 million people yearly [2]. Research shows that the majority of adverse events can be avoided, resulting in lives saved, avoidance of suffering, and cost savings.

Adverse events related to drugs and medication errors are common occurrences, impose substantial costs to the healthcare system, and are clinically relevant [3]. Considering the shortcomings of the Brazilian health system, such as lack of funds, low remuneration, inadequate technical preparation of workers, and inadequate technology, it can be assumed that medication errors are a major cause of patient harm.

Different approaches are being implemented to improve patient safety. One of these is to empower frontline workers to innovate and improve compliance in different business sectors [4, 5]. This is called Positive Deviance (PD) and has been applied in the healthcare setting to improve hand hygiene compliance, reduce methicillin-resistant *Staphylococcus aureus* infections, and reduce bloodstream infections in an outpatient hemodialysis center [5]. Positive Deviance (PD) is a tool for improving processes using the suggestions of those involved. In this case, the healthcare personnel (doctors, pharmacists, nurses, nursing assistants, pharmacy and nursing technicians, pharmacy assistants, and warehouse assistants, etc.) in their daily routine, discover small details that make a difference in patient safety. PD is a behavioral change approach based on the premise that in any situation some individuals faced with the same type of problem have innovative ideas and are able to find the best solution [6].

Therefore, this study aimed to analyze medication errors and perform an intervention using Positive Deviance to reduce them in a tertiary care hospital.

## Methods

This study was approved by the Ethics Committee of the Instituto Israelita de Ensino e Pesquisa Albert Einstein and carried out in the inpatient setting, operating room and Emergency Department of a tertiary care hospital with 625 beds. The requirements for informed consent were waived by our IRB in accordance of the Code of Federal Regulation and of the Privacy Rule. It was performed in a prospective manner between the years 2011 and 2013. Medication errors are reported in an electronic system called *SIEN - Sistema Einstein de Notificação* (ENS - Einstein Notification System). The SIEN was developed internally, and allows anonymous reporting of errors by all workers through the hospital intranet. These errors are reported in a standardized manner and are stored in a database. For each report, the managers of the area or areas involved in the error automatically receive an email with a copy of the report.

The errors were classified using the National Coordinating Council for Medication Error Reporting and Prevention (NCC MERP) to standardize the taxonomy and classification of errors (Table 1) [7–10]. Drugs were classified as either high-alert medications or not. In this study, chemotherapy drugs, anticoagulants, insulin, and morphine were considered high-alert medications.

**Table 1:** Classification of medication errors according to the National Coordinating Council for Medication Error Reporting and Prevention [10]

Error class	Definition	
A	No error	The circumstances or events have the capacity to cause error
B	Error, no harm	An error occurred but did not reach the patient
C		An error occurred, reached the patient but did not cause harm
D		An error occurred, reached the patient and required monitoring to confirm that it resulted in no harm to the patient and/or required intervention to preclude harm
E	Error, harm	An error occurred, that may have contributed to temporary harm and required intervention
F		An error occurred, that may have contributed to or resulted in temporary harm, and required initial or prolonged hospitalization
G		An error occurred that may have contributed to or resulted in permanent harm
H		An error occurred that required intervention necessary to sustain life
I	Error, death	An error occurred that may have contributed to or resulted in the patient’s death

The errors were analyzed in three time periods: Phase I: January-December 2011, which was the control phase; Phase II: January-December 2012, which was the manager intervention phase; and Phase III: January-December 2013, which was the healthcare worker intervention phase. In Phases II and III of this study, Positive Deviance [5, 6] was used to reduce medication errors.

In Phase I of this study medication errors classified “A” to “C” according to the NCC MERP were analyzed by the work team at the place where the error occurred. Errors classified as “D” were analyzed in conjunction with the Board of Healthcare Practice, Quality, Safety, and Environment, and for the errors classified as “E” and above, the errors were analyzed using root cause analysis.

In Phase II, the Positive Deviance method began to be used to analyze errors that reached the patient. Meetings were held with middle managers, including nurse managers, nursing and pharmacy coordinators, and medical managers. In these monthly meetings, any errors classified from “C” or greater on the NCC MERP scale, which are those medication errors that reached the patient, were discussed, and procedures for the mitigation of medication errors were proposed and subsequently implemented. The meetings were coordinated by the pharmacy manager and strong procedures were implemented to decrease medication errors, such as double-checking between the nursing technician and the nurse at the time of administering insulin, checking patient identity, the medication, and the dose prepared. Another procedure was to double-check the preparation and planning of the infusion pump for vasoactive drugs. The reading of the medical prescription by the nursing technician and the nurse at the beginning and end of each duty shift in order to answer any questions about the drugs, reading the medical prescription from left to right, and, at the end of shift, also check whether all medications had been properly administered and checked. Morphine prescriptions by 1:10 dilution were prohibited and only prescriptions by dosage were allowed. Morphine doses above 6 mg were required to be validated by the pharmacy. A working group called the Medication Safety Group (MSG) was created for technical level pharmacy workers and another one specifically for pharmacists. These groups met once a month, separately, to discuss and propose changes in the process to prevent medication errors.

In Phase III, the PD method continued to be used and a campaign called ‘Safe Route in the Medication Process’ was initiated. In this campaign, Medication Safety Groups (MSGs) were formed in all hospital units. These groups included pharmacists, nurses, and nursing and pharmacy technicians. The units’ MSGs held meetings every 2 weeks led by the clinical pharmacist of the unit. In these meetings, the unit errors were discussed and proposals were drawn up to mitigate the medication errors.

Overall, there were 21 MSGs, totaling 402 workers who met every 2 weeks to propose and implement procedures to decrease medication errors in their units. In this phase, management did not participate in the meetings with the intention of leaving the staff to make their own analyses and proposals. It was up to management to filter and enable the implementation of the proposed ideas.

For this campaign, a banner of 1.60 m by 1.20 m was made with the campaign logo “Safe Route in the Medication Process”, which was hung up in the corridor of each hospital ward, visible to all healthcare workers, patients, and visitors. On this banner the monthly rate of medication errors was recorded for that hospital ward, the comparison of the rate of medication errors that had reached the patient along with the goal of the hospital, and the agreed upon reduction target of the ward. Near miss reports, classified as “A and B” medication errors, were also compared and their reporting was encouraged. There was also space on the banner for people to write suggestions for improving processes.

In this campaign, awards were given to the units that had reduced the rate of medication errors classified as “C” onwards, the group that had implemented the most interventions, and the group that had implemented the largest number of interventions in the entire hospital.

To determine the rate of serious adverse events (SAE) related to medication, ie, classes E through I, the number of SAEs was divided by the number of patient-days and multiplied by 100,000.

### Statistical analysis

The descriptive analysis of the errors was performed using absolute and relative frequencies for each of the phases. In order to check for trends in reported error rates in each of the phases, ARIMA (autoregressive integrated moving average) models for time series with intervention were adjusted for each error.

The Pearson correlation was estimated to check whether there was any relationship between the rate of errors and the inpatient bed occupancy rate.

The critical care units (Adult Intensive Care Unit and Step-down, neurological and cardiac units), medical-surgical unit, maternity, neonatal ICU, newborn nursery, and oncology inpatient units were studied. The study phases were compared with regards to shift, professional category, type of prescription (manual or electronic), class of the medication, the team that committed the error (accountability), time, phase of care (including prescription, transcription, validation by the pharmacist, drug administration, and monitoring), gender, and age categories. Results of the models were presented as estimates and the 95 % confidence intervals for increases (or decrease) after each intervention. In order to check for any difference between the phases regarding qualitative variables, we used Fisher’s exact tests or Chi-squared tests. All analyses were performed using the statistical package R (R Core Team, 2013). The ARIMA models were adjusted using the Forecast [11] package.

## Results

The errors occurred mostly in the medical-surgical unit, which accounted for 2713 (68.0 %) cases from a total of 3991. In Phase I, in the medical-surgical unit there were 771 (73.1 %) medication errors out of a total of 1054; in Phase II, there were 792 (71.1 %) out of a total of 1098; in Phase III, there were 1150 (62.0 %) out of a total of 1839. Despite the increase in the number of reported errors (Table 2), a decrease in the proportion of errors of 11.1 % can be seen when comparing Phase III with Phase I and of 9.7 % when comparing Phase III with Phase II.

**Table 2:** Medication error rates over the study phases

	Phase I - 2011	Phase II - 2012	Phase III - 2013
Prescribed medications	2,365,231	2,591,863	2,813,633
Medication errors reported	1054	1105	1854
Medication error rate (%)	0.04	0.04	0.07
Patient-days	188,242	194,353	198,534
Medication-related serious adverse events (SAE)	18	9	5
Percent of medication errors that are SAE (%)	1.7	0.8	0.3
Rate of SAE (per 100,000 patient-days)	9.6	4.6	2.5

As for time of the day, the morning (0700 to 1300 h) period showed the greatest number of errors in the first two phases of this study: 348 (33.0 %) out of a total of 1054 in Phase I and 424 (38.4 %) out of a total 1105 in Phase II. In Phase III, the night shift had the greatest number of errors, with 874 (47.1 %) out of 1839 errors.

The errors were also classified into five stages: medical prescription, transcription, validation of the prescription by the pharmacist, administration, and monitoring. In the three phases combined, the greatest number of errors occurred during the administration of medications, with 35.5 % (374 of 1054 errors) in Phase I, 43.1 % (476 out of 1105 errors) in Phase II, and 55.6 % (1030 out of 1854 errors) in Phase III (Table 3).

**Table 3:** Descriptive epidemiology of medication errors

	Phase I - 2011 (*n* = 1054) %	Phase II - 2012 (*n* = 1105) %	Phase III - 2013 (*n* = 1854) %	P**
Location:				*p* < 0.001
• Operating room	1.2	1.8	1.5	
• Medical surgical unit	73.1	71.7	62.0	
• Pharmacy	0.0	0.1	4.6	
• Step Down Unit	10.9	8.3	20.7	
• Emergency Department	0.0	2.6	0.0	
• Intensive Care Unit	14.7	15.5	11.2	
Time:				*p* < 0.001
• Morning (7 am to 1 pm)	33.0	38.4	30.9	
• Not identified	13.4	0.2	0.8	
• Night (7 pm to 7 am)	23.5	31.0	47.1	
• Afternoon (1 pm to 7 pm)	30.1	30.4	21.3	
Order entry type:				*p* < 0.001
• Electronic	38.4	40.4	73.7	
• Manual	61.6	51.4	26.3	
• Not Prescribed	0.0	8.2	0.0	
Error class:				*p* < 0.001
• A	29.2	14.1	2.2	
• B	25.8	34.8	65.1	
• C	35.7	35.2	27.5	
• D	8.0	13.8	4.6	
• E, F, H, G or I	1.3	2.1	0.5	
Error accountability:				*p* < 0.001
• Nursing	46.4	48.5	58.7	
• Pharmacy	19.4	20.1	26.9	
• Physician	32.4	22.9	10.8	
• Other	1.7	8.5	3.6	
Phase of medication use where error occurred:				*p* < 0.001
• Administering	35.5	43.1	55.6	
• Dispensing	19.6	24.3	22.7	
• Monitoring	3.4	0.9	0.2	
• Prescribing	34.5	23.8	11.7	
• Transcribing	6.9	8.0	9.9	

**Fisher’s exact test or Chi-square test

According to the NCC MERP classification, there was a significant increase of near miss reports in Phase III. Near miss reporting was encouraged by the “Safe Route in the Medication Process” campaign. The “A” and “B” classification represented 67.3 % (1248) of notifications from Phase III *versus* 48.9 % (907) from Phase II and 55.0 % (578) from Phase I.

The types of errors, listed as either high-alert medication or not, are described in Table 4. The majority of the errors that were not considered high-alert medication errors were missed administration of medications (693, 22.3 %), administered at the wrong time (483, 15.5 %), wrong medication (409, 13.2 %), or wrong dosage (316, 10.2 %). For the cases of high-alert medications, the majority of errors were administration at the wrong time (150, 16.6 %), followed by prescription error (140, 15.5 %), wrong medication (112, 12.4 %), and wrong dosage (110, 12.2 %). The volume of errors reported increased from 1054 in Phase I to 1854 in Phase III.

**Table 4:** Types of errors for high-alert medications from 2011 to 2013

	High-alert medication		*p*
	No	Yes	
	*N* = 3108	*N* = 905	
	*n* (%)	*n* (%)	
Wrong Time	483 (15.94)	150 (16.93)	0.45^b^
Prescription Error	230 (7.59)	140 (15.80)	<0.001^b^
Wrong medication	409 (13.50)	112 (12.64)	0.54^b^
Wrong dosage	316 (10.43)	110 (12.42)	0.09^b^
Medication not administered	693 (22.87)	85 (9.59)	<0.001^b^
Administration error	229 (7.56)	60 (6.77)	0.45^b^
Wrong infusion time	54 (1.78)	59 (6.66)	<0.001^b^
Absence of prescription	66 (2.18)	27 (3.05)	0.13^b^
Error in preparation	58 (1.91)	26 (2.93)	0.06^b^
Wrong Patient	128 (4.22)	26 (2.93)	0.09^b^
Error in acquisition	75 (2.48)	21 (2.37)	0.87^b^
Wrong dilution	34 (1.12)	19 (2.14)	0.02^b^
Wrong route	78 (2.57)	19 (2.14)	0.48^b^
Unauthorized administration	31 (1.02)	11 (1.24)	0.57^b^
Wrong administration technique	9 (0.30)	7 (0.79)	0.07^a^
Wrong treatment duration	9 (0.30)	6 (0.68)	0.12^a^
Wrong date	11 (0.36)	5 (0.56)	0.38^a^
Allergy not considered	23 (0.76)	4 (0.45)	0.33^b^
Illegible	30 (0.99)	3 (0.34)	0.06^b^
Wrong paperwork	31 (1.02)	3 (0.34)	0.05^b^
Prior history of allergy	89 (2.94)	3 (0.34)	<0.001^b^
Monitoring	5 (0.17)	3 (0.34)	0.39^a^
Absence of checking	4 (0.13)	2 (0.23)	0.62^a^
Dispensing error	8 (0.26)	2 (0.23)	>0.99^a^
Not Identified	2 (0.07)	1 (0.11)	0.54^a^
Inadequate actions of patient/family	3 (0.10)	1 (0.11)	>0.99^a^

^a^Fisher’s Exact Test; ^b^Chi-square test

**Fig. 1:**
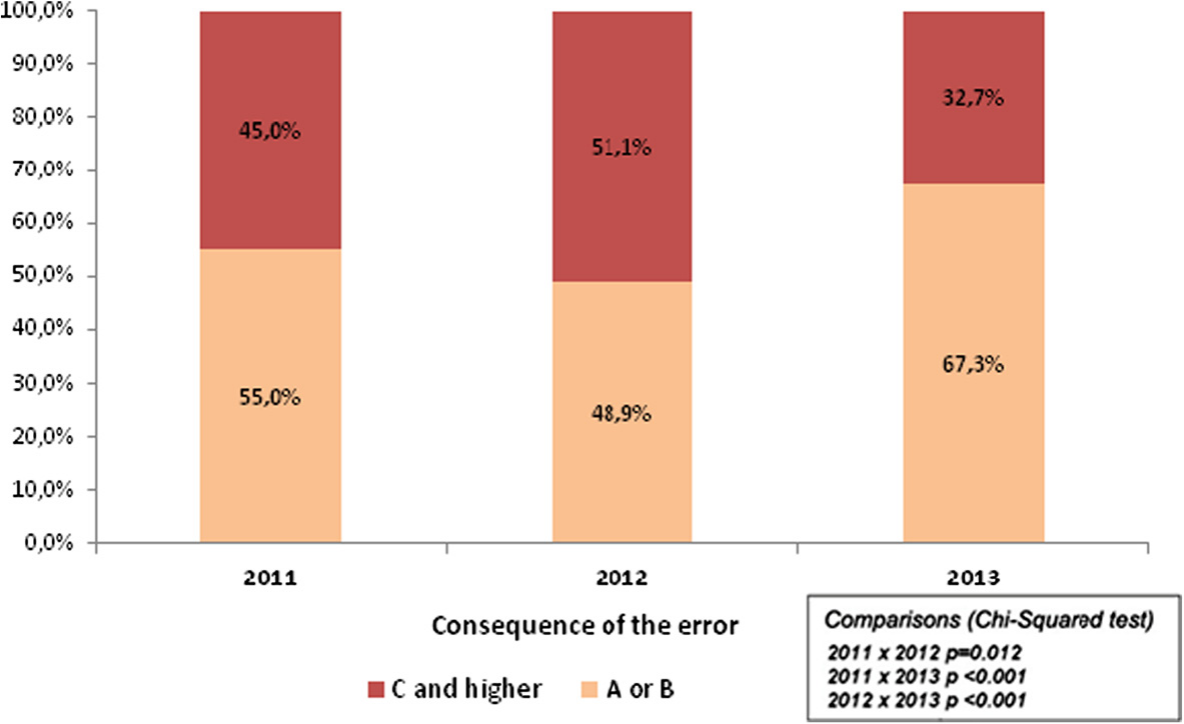
General description of the consequences of errors

The “C and D” classification represented 32.7 % (606 out of 1854) of notifications from Phase III. In Phase II, the “C and D” classification represented 51.1 % (565 out of 1105), and in Phase I, for that same classification, there were 45 % (474 out of 1054). There were 123 interventions implemented in Phase III and, despite the increase in the number of reported errors, it was observed that from classification “C” onwards, according to the NCC MERP, the percentage diminished in relation to previous years (*p* = 0.012 for 2011 vs. 2012; *p* < 0.001 for 2011 vs. 2013 and *p* < 0.001 for 2012 vs. 2013), as shown in Fig. 1.

In order to study the evolution of the error rate, time series models were used to evaluate the impact of the interventions. For the error rate (general and by classification), an ARIMA model showed that there was a low and ascending relationship between the interventions and reports of errors. At each intervention, an increase was expected in the reported error rate of 0.16 (95 % CI, 0.24 to 0.39).

For error types “A and B”, the adjusted model showed that there was a moderate and ascending relationship between the interventions and error reports. At each intervention, an increase was expected in the reported error rate of 0.19 (95 % CI, 0.13 to 0.25). For error types “C to I,” there was a descending relationship between the interventions and error reports. At each intervention, a decrease was expected in the notified error rate of 0.12 (95 % CI, 0.18 to 0.07). For error types “D to I,” we observed a positive and ascending relationship between the interventions and error reports. At each intervention, an increase was expected in the reported error rate of 0.02 (CI 95 % 0.01 to 0.03).

**Fig. 2:**
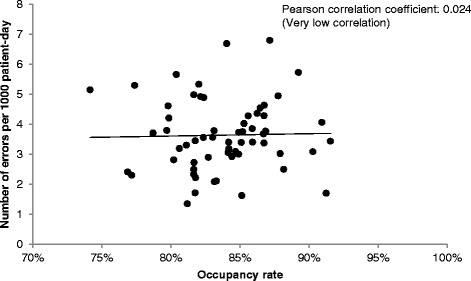
Correlation between the frequency of medication errors and bed occupancy from 2011 to 2013

The correlation between the number of medication errors and the bed occupancy rate was very low (Pearson correlation coefficient 0.024; Fig. 2).

Overall, there were 123 interventions implemented. Among those (Table 5), we highlight the double-checking of vasoactive drugs, insulin, multi-dose psychoactive medications (oral solutions and drops), and medications administered via infusion pump at the time of administration. Another intervention was changing the packaging of sterile petroleum jelly from ampoules to refillable bottle to prevent erroneous intravenous administration, and another was labeling glycerin enema as rectal use only. A reminder was also implemented to perform medication reconciliation when patients were transferred between units.

**Table 5:** Top 20 interventions of 123 interventions implemented

Top 20 interventions implemented	
1. double-checking of vasoactive drugs	11. purchase carts for nurses to prepare medicines at patients’ bedsides
2. double-checking of insulin	12. reminder implemented to avoid loss of drugs in the transfer between units
3. double-checking of multi-dose psychoactive medications	13. morphine doses greater than 6 mg prepared exclusively by pharmacy
4. double-checking of medications administered via infusion pump at the time of administration	14. sound and look alike drugs stored in different places
5. packaging of sterile petroleum jelly from ampoules to refillable bottle to prevent erroneous intravenous administration	15. high box use (capital letters) in the bar code identification and in the medical prescription and in the prescription for the medications sound alike look alike (ETILEfrine vs EPINEPHrine)
6. reminder was implemented to perform medication reconciliation when patients were transferred between units	16. Require prescription of drugs in doses rather than unit (tablet, vial, ampoule)
7. double checking medical prescription between nurses during shift changes	17. patient education for the conference identification bracelet with prescription at the time of drug administration
8. standardization in the medical team to change drug infusion rates	18. reinforcement for nursing staff for the immediate return of drugs suspended
9. reminder implemented for patient allergy insertion into the prescription form	19. change the color label for compounded drugs
10. insertion into prescription by pharmacist the diluent volume and rate of infusion of medications that may cause phlebitis	20. challenge units to increase reporting of medication errors

## Discussion

Reduction in medication errors is possible using the PD approach. In traditional approaches, senior leaders make the decisions, middle managers implement them, and workers execute them while rarely being engaged in those solutions. The PD approach allows frontline workers to decide how the changes should be performed by promoting the participation of all those involved, thereby promoting the resolution of problems between peers. It is up to those in upper and middle management to filter the workers’ ideas and to remove the barriers to the implementation of the best practices. In truth, the PD tool is the reversal of the work pyramid, and the leaders only provide support to the workers [6].

In Phase III, a Medication Safety Group in each hospital unit was created and two pharmacy MSGs were maintained. In this phase, the management was removed from the meetings that were held using the Positive Deviance method, so that the participants could be more collaborative without feeling embarrassed to contribute with new ideas.

In this phase, there were more than 400 workers participating in the meetings of 36 MSGs throughout the hospital. The “Safe Route in the Medication Process” was a great campaign, and it rewarded the implemented ideas that increased patient safety and also rewarded those units where errors had decreased significantly. In Phase III, out of 237 procedures proposed, 123 were implemented. Among those, we can highlight the separated storage areas of medicines with sound-alike, look-alike names, spellings, and packaging; validation of orders for analgesics; the increase in hand-off time when transferring patients; the enhanced annotation of any allergy in the patient’s medical prescription; and the disallowing of any erasure on the medical prescription of dilution, volume, and time of infusion for injectable medications in the medication order. In this phase, there was a decrease of 45 % (9 in Phase II and 5 in Phase III) of serious adverse events with medications when compared with Phase II. There was a decrease of 72 % (18 in Phase I and 5 in Phase III) when compared with Phase I.

In Phase III, there was also an increase of 152 % (380 in Phase II and 959 in Phase III) of reports of near miss medication errors, demonstrating the concern of the entire health team.

In this study, the locations, shift, type of prescription, classification, and “accountability” were compared for each of the phases, as well as whether the drug involved in the error was a high-alert medication. When compared with another study [1], the findings in Phase III of the present study were not very different. The highest percentage of error – 55.6 % (1030 out of 1854) was shown to be during administration, followed by 22.7 % (421 out of 1854) during dispensing, and 11.7 % (216 out of 1854) while prescribing, 9.9 % (184 out of 1854) during transcribing and validating the medical prescription, and 0.2 % (3 out of 1854) during monitoring.

In this study, 4013 medication errors were evaluated, and overall the prescription errors, errors in transcribing the medical prescription, errors by the pharmacist in validating the prescription, dispensing errors, administration errors, and monitoring the use of medications were similar to other studies [1, 2, 6].

### Limitations

The main limitation of the present study was that the data was obtained through a passive surveillance system, where the reporting is voluntary and anonymous. It is known that errors are underreported worldwide [1, 3, 9] in spite of the culture of error reporting implemented at the Hospital Albert Einstein, which may in some way limit the direct comparability with other studies that report the rate of medication errors. Another factor is that in the institution studied, the errors in the “A, B, and C” categories may be underreported, but due to the culture, category “D to I” errors are rarely under-reported [12–15]. What contributes to the increase in reporting is that the SIEN is an anonymous system, non-punitive, and easy to use.

Although the reporting of medication errors represents opportunity for improving processes, due to the system being anonymous, it is not possible to validate the reporting. Previous studies on medication errors have shown a high degree of reliability, which increases the reliability of the present data because all errors recorded in the SIEN are classified according to the NCC MERP in order to standardize the reporting [16, 17]. In cases of doubt about any report, all notifications with a “D to I” classification are analyzed on the spot.

Although our surveillance system may have limitations, we believe it is better than actively searching for medication errors in medical records and searching for observational errors, which can lead to even greater inaccuracies. Direct observation provides the most accurate error rate, however, it may induce the Hawthorne effect [15] and make the healthcare workers who are being observed develop a positive change in their behavior in relation to the goals of observation [17–20].

## Conclusions

Positive Deviance proved to be effective, particularly when there is the participation of involved healthcare professionals, as was the case in Phase III of the present study.

Medication errors are still of great concern, accounting for serious adverse events in patients, and should always be monitored. Procedures to mitigate them should be implemented with the participation of frontline workers. In this study, we also demonstrated that with the greater the number of events that were reported, the greater the number of interventions were implemented, and there was a fall in the rate of the most serious errors. The reporting of errors and the control procedures implemented are much more effective when disclosed to the healthcare workers, as evidenced in Phase III of this study. For this reason, error disclosure to workers should be mandatory.

## Abbreviations

ARIMA, autoregressive integrated moving average; ENS, Einstein Notification System); ICU, intensive care unit; IRB, Intitutional Review Board; MSG, Medication Safety Group; MSGs, Medication Safety Groups; NCC MERP, National Coordinating Council for Medication Error Reporting and Prevention; PD, Positive Deviance; SAE, serious adverse events; SIEN, Sistema Einstein de Notificação
